# Population health management through human phenotype ontology with policy for ecosystem improvement

**DOI:** 10.3389/frai.2025.1496937

**Published:** 2025-08-01

**Authors:** James Andrew Henry

**Affiliations:** Institute of Biomedical Sciences, London, United Kingdom

**Keywords:** population health management, safety, security, predictive health, precision care, classifications, human phenotype ontology policy

## Abstract

**Aim:**

The manuscript “Population Health Management (PHM) Human Phenotype Ontology (HPO) Policy for Ecosystem Improvement” steward safe science and secure technology in medical reform. The digital HPO policy advances Biological Modelling (BM) capacity and capability in a series of fair classifications. Public trust in the PHM of HPO is a vision of public health and patient safety, with a primary goal of socioeconomic success sustained by citizen privacy and trust within an ecosystem of predictor equality and intercept parity.

**Method:**

Science and technology security evaluation, resource allocation, and appropriate regulation are essential for establishing a solid foundation in a safe ecosystem. The AI Security Institute collaborates with higher experts to assess BM cybersecurity and privacy. Within this ecosystem, resources are allocated to the Genomic Medical Sciences Cluster and AI metrics that support safe HPO transformations. These efforts ensure that AI digital regulation acts as a service appropriate to steward progressive PHM.

**Recommendations:**

The manuscript presents a five-point mission for the effective management of population health. A comprehensive national policy for phenotype ontology with Higher Expert Medical Science Safety stewards reform across sectors. It emphasizes developing genomic predictors and intercepts, authorizing predictive health pre-eXams and precise care eXams, adopting Generative Artificial Intelligence classifications, and expanding the PHM ecosystem in benchmark reforms.

**Discussion:**

Discussions explore medical reform focusing on public health and patient safety. The nation's safe space expansions with continual improvements include stewards developing, authorizing, and adopting digital BM twins. The manuscript addresses international classifications where the global development of PHM enables nations to choose what to authorize for BM points of need. These efforts promote channels for adopting HPO uniformity, transforming research findings into routine phenotypical primary care practices.

**Conclusion:**

This manuscript charts the UK's and global PHM's ecosystem expansion, designing HPO policies that steward the modeling of biology in personal classifications. It develops secure, safe, fair, and explainable BM for public trust in authorized classifiers and promotes informed choices regarding what nations and individuals adopt in a cooperative PHM progression. Championing equitable classifications in a robust ecosystem sustains advancements in population health outcomes for economic growth and public health betterment.

## 1 Introduction to science and technology-driven population health management

Population Health Management (PHM) Human Phenotype Ontology (HPO) Policy for Ecosystem Improvement is a national programme stewardship proposal for the UK Department of Health and Social Care that commission quality in an integrated care ecosystem (GOV.UK, 2024f). A PHM mission for HPO, biology, models, and classifiers is an informatic data-driven approach integrating scientific data themes in multi-omics, health-determining factors, imaging, and behavioural assessments as digital twin technologies advance wellbeing (Mohr et al., 2024). The author advocates public inclusiveness and stakeholder engagement in Biological Modelling (BM) with an HPO policy-in-practice that develops, authorizes, and adopts predictors and intercepts classifiers as the medical norm (van Ede et al., 2023).

PHM of an ageing population, specialist referrals, hospital readmissions, and avoidable emergency visits has societal health and economic costs that need HPO identification and management (Theodorakis et al., 2025; Barrio-Cortes et al., 2021). The author redefines adverse events to address poor health epidemics with genomic screens that deliver an ontology-based approach (Kumah, 2025). PHM encounters obstacles to pathology segmentation for common rare diseases, which affect 6% of citizens, while non-communicable disorders in chronic conditions account for more than 80% of primary care consultations (GOV.UK, 2021). Meanwhile, health systems face delays and diversity in oncology detection or therapy, while prescription medications are unsuitable for patients, necessitating data-driven innovations for parity (Wu et al., 2024; Kimura et al., 2022).

National PHM of quality lives are shaped by genomic data science and AI technology, which steward public health and improve social care while enhancing patient safety in value-based primary care use cases (Department of Science, 2023; Department of Health and Social Care, 2022). HPO policy development and implementation for societal benefits cluster advanced scientific data themes with algorithms that personalize lifecycle health journeys by ensuring BM and HPO accessibility in robust data shares and security protocols for a privacy-structured intelligence ecosystem (Søe, 2023). As federated learning scales AI-driven predictive analytics and precision tools in private classification, the multi-omics and socioenvironmental factors envision a vision of medical reform (Ren et al., 2023).

## 2 Background to HPO organisation and policy development

**Figure 1:**
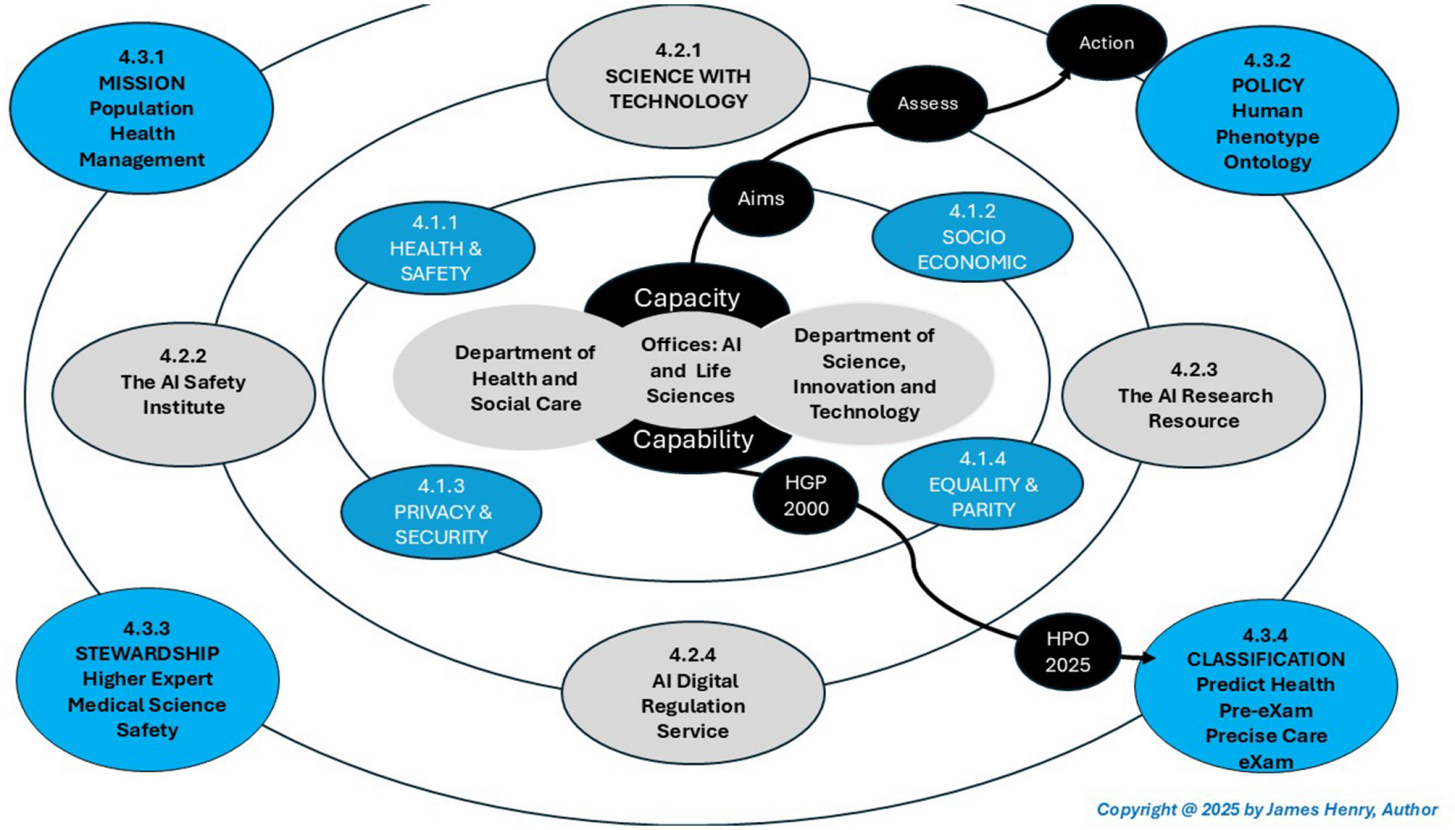
UK ecosystem, safe space.

Figure 1 illustrates a quarter century of HPO organisation and policy development for science and technology in an ecosystem expansion from the Human Genome Project to the global classifications proposed for genomic disease prediction (pre-eXams) and intercepts (eXams). Initially focused on Mendelian diseases, HPO differentiates annotations within the Global Alliance for Genomics in Health, as we evolve clinical justification in the classifications proposed (Global Alliance for Genomics and Health, 2021). Furthermore, the Monarch Institute has evolved HPO personalized applications in a digitised vocabulary, enabling tools like exomiser and phenomizer to unify personalized risk stratification of abnormalities with disease segmentation, which explores accurate intercepts (Human Phenotype Ontology, 2024).

The Department of Health and Social Care benefits from the coordination, accountability, and stewardship of this PHM mission through the excellence of the integrated care ecosystem (Gov.UK and Department of Health Social Care, 2025). Health and Social Care navigates PHM ethics, scientific data sharing, BM funding, HPO privacy and security whilst genomics AI network and AIDRS stewards align research, regulation, commission, and governance of Generative Artificial Intelligence (Gen AI) development and adoption (National Health Service, 2024a). In addition, PHM requires updates to the Care Quality Commission (CQC) for safe space assessments in a national integrated care ecosystem with progressive BM in standard HPO policy for citizen genomics digital twins (Care Quality Commission, 2024).

The Department of Science, Innovation and Technology identified Areas of Research Interest (ARI) in which groups may engage the PHM of HPO (GOV.UK, 2024b). The UK Science and Technology Network (STN) and Science, Innovation, and Growth Group (SIG) advance the PHM landscape, maximising research and development investments to align government priorities in genomic hubs, life sciences, biotechnology, pharmaceuticals, quantum computing, and BM engineering (GOV.UK, 2025f). The UK Offices of Life Sciences and AI with STN and SIG connect government with academia and industry with science and technology for medicine as PHM deploys socioeconomic success over the coming decade (GOV.UK, 2024c,e).

## 3 Overview of advancing biological models capacity and capability

Figure 1 expands the PHM mission in an integrated ecosystem with an AIDRS HPO policy for Higher Expert Medical Science Safety (HEMSS) in genomics predictors and target intercepts as personalized developments authorize classifiers that steward HPO with BM adoption. This overview of advancing BM capacity and capability considers three points:

1. **Scientific data themes** commence in the genomics network alignment with the medical service “hubs and test directories,” the royal college of pathologists' “end-to-end pathology” workflows, and biobanks “data repositories,”; as well as the national pathology imaging co-operative's “alignment” in imaging practices (N England, 2024; Myers et al., 2021; UK Biobank, 2019; National Pathology Imaging Cooperative., 2025). These key data shares exemplify the integration of healthcare informatics and management systems society (HIMSS) operability (HIMSS, 2021). HIMSS adoption advice for PHM maturity and volunteering BM science-themed data has nations institutionalize reform (HIMSS, 2021; GOV.UK, 2025d).
2. **Technological AI** integrates the safety/security institute (AISI), research resource (AIRR), and digital regulation service (AIDRS), which contributes to a progressive PHM ecosystem (GOV.UK, 2025d; UK Research and Innovation, 2025; NHS England and NHS Transformation Directorate., 2025). These entities align HEMSS, ensuring ethical and scientifically robust practices which benefit from developing, authorizing, and adopting national BM hybrid AI practices (GOV.UK, 2025d; UK Research and Innovation, 2025; NHS England and NHS Transformation Directorate., 2025). Incorporating AI-driven informatics and multi-omics predictive tools enhances PHM's capabilities to deliver personalized healthcare solutions, addressing challenges in rare and major disease segmentation, which reduces misdiagnoses for accurate intercepts (Stark, 2023; Jayasinghe et al., 2024).
3. **An HPO policy** steward's robust BM infrastructure capability for PHM as cross-discipline hybrid AI practice research pangenome specificity within multi-omics for HPO characterisation (Huang et al., 2024). Scientific data capacities with stakeholder engagement and public inclusiveness in fairness build PHM trust and public/partner confidence (Kerasidou, 2023). Federated learning and quantum computing for BM capacity and HPO capabilities in health service delivery and public health expand and translate ecosystem outcomes (Yapar, 2024; Gupta et al., 2024). Capable PHM stewards review' scientific data and scale classical gen AI capacity in an ecosystem of genomics predictors and life science intercepts (Sas, 2025).

## 4 Population health management for our future wellbeing

Figure 1 expands safe space, which centers on authorities for scientific capabilities and technological capacity that activate the PHM of HPO. Figure 1 shows the ecosystem structure from central government departments, which tiers the aims and assessments whilst actioning the PHM mission through AIDRS Genomic Network HPO policy for HEMSS in the stewardship of classifications. An overview of the manuscript sections includes.

**Public health aims**. The manuscript articulates national, and citizen aims, emphasizing public health and safety, promoting socioeconomic success, safeguarding privacy and security, and ensuring equality and parity through BM. Public inclusiveness and stakeholder engagement highlight the ethical and practical implementation of ecosystem safe spaces that tier government capacity and capability for national aims, as depicted in Figure 1.

**Figure 2:**
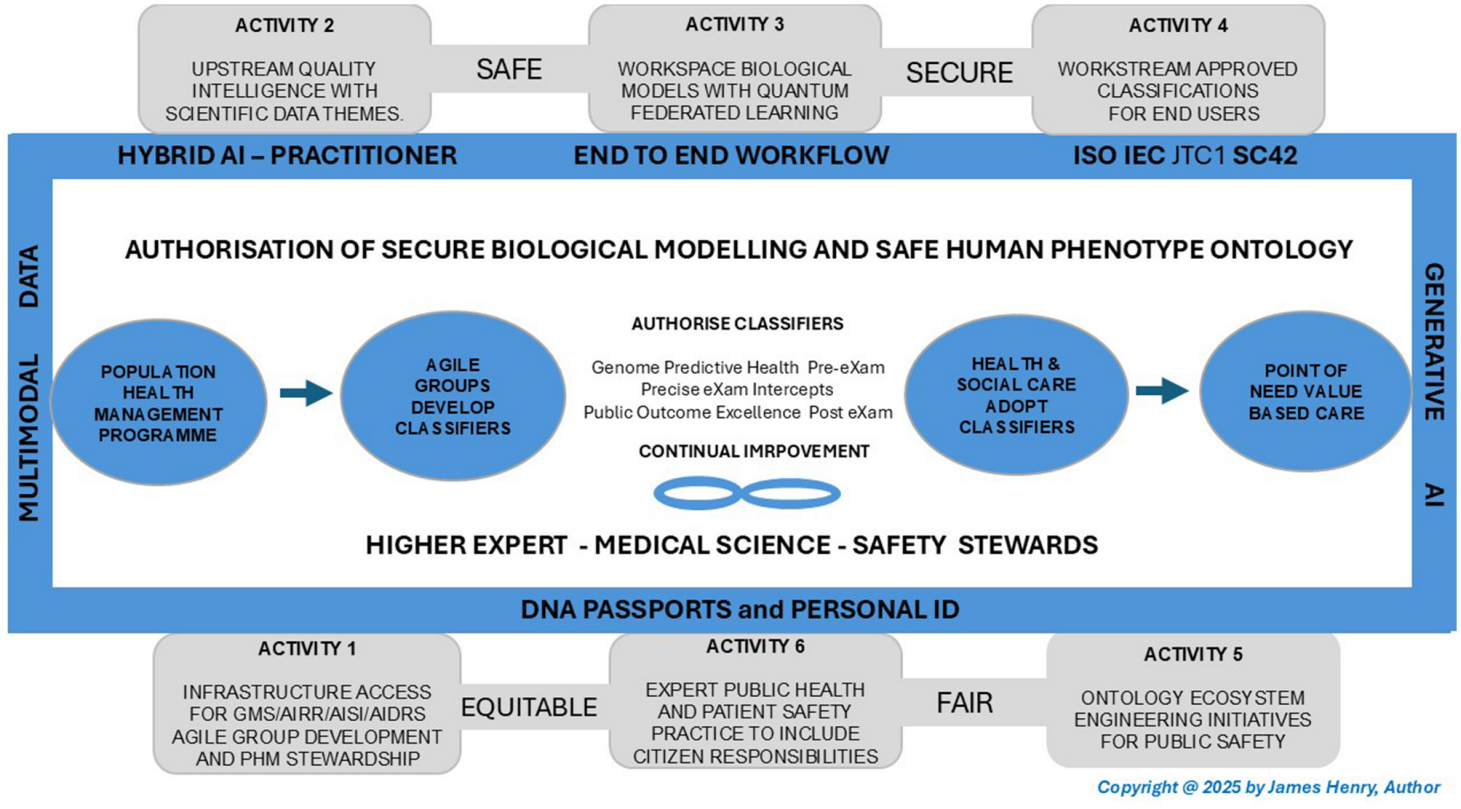
Population health management, human phenotype ontology continuous improvements.

**Medical phenotype assessments**. The author details science and technology evaluations with roles for the GMS, AISI, AIRR, and AIDRS to ensure that BM classifiers are ethically assessed and regulated. Evaluating security and safety is with federated learning, quantum intelligence, and generative pre-trained transformer 5 (GPT-5) as PHM develops an ecosystem of HPO improvements, as depicted in Figure 2. This section on “assessment” outlines key mechanisms for evaluating an HPO policy.

**Figure 3:**
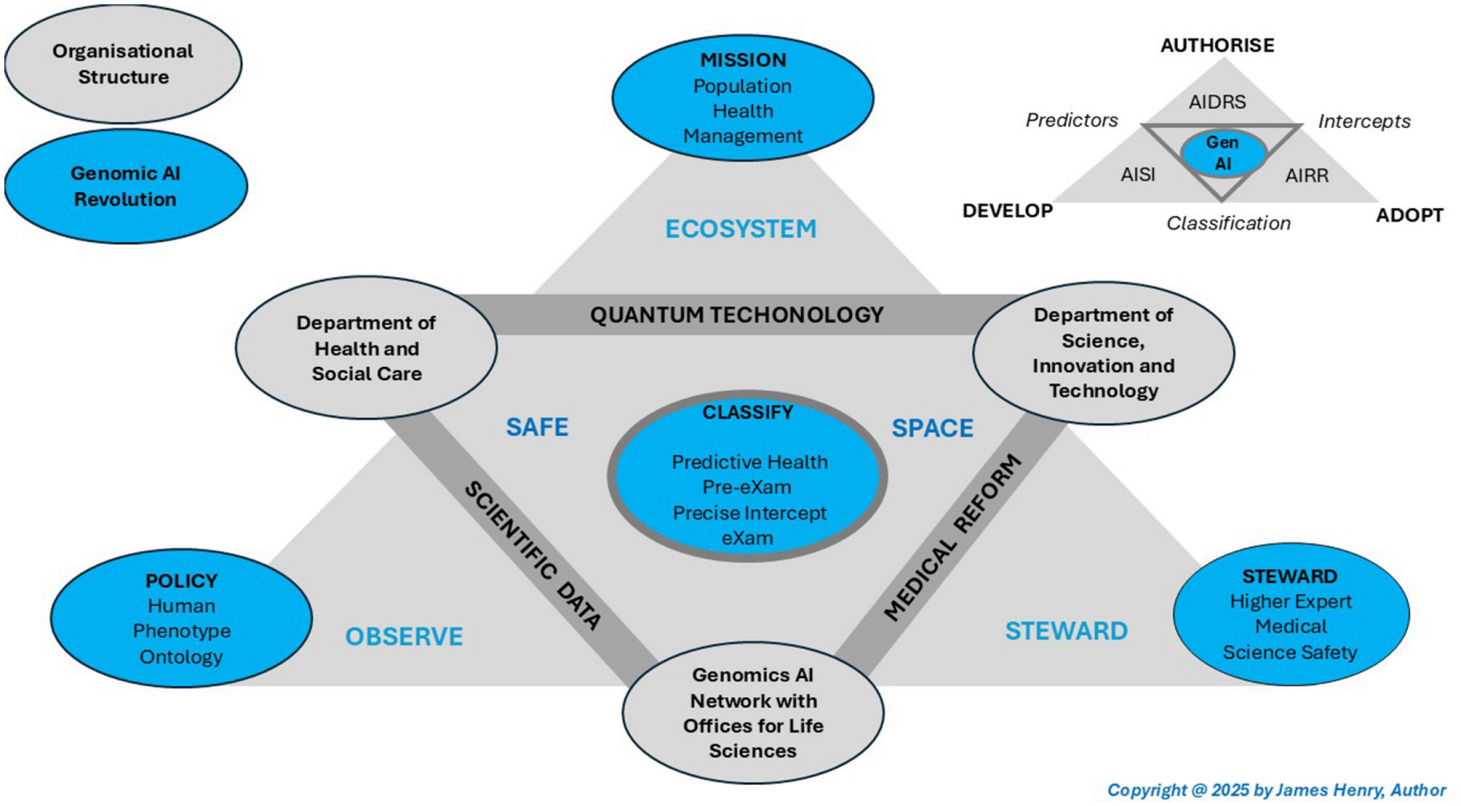
Human phenotype ontology policy with higher expert medical science safety which Steward classifications.

**Population health proposition**. The article postulates a PHM five-point mission and HPO policy with Higher Expert Medical Sciences Safety (HEMSS) stewardship of public inclusivity and stakeholder engagement to develop, authorize and adopt classifiers. The principal ecosystem action is to predict health in pre-eXams for precise care through eXams with Gen AI [X] classifications, where “[X]” refers to specific predictor and intercept features that cluster in data, central to the transformational reforms triangulated and depicted in Figure 3.

**Discussions with stakeholders**. The reader engages with discussions that surround HPO policy from multiple perspectives that steward BM transformation in classifications. Digital evaluation of impacts on citizen wellbeing and economic growth debate stewarding with key discussion points for public health, patient safety, and equity. The conclusion reviews the sections to affirm the PHM mission reform for medicine with HPO policy as HEMSS steward classifications.

### 4.1 Population health management aims for human phenotype ontology

PHM develops BM value-based care that features scientific data themes in authorized use case classifiers that stream predictors and intercepts (The Association of Clinical Biochemistry and Laboratory Medicine, 2023). As detailed in Sections 4.1.1–4.1.4, this approach supports a national vision for PHM that aims to characterise HPO. The mission for public health and patient safety that sustains socioeconomic success with citizen privacy, data security, and parity in outcomes is described in Tables 1–4, which interlink assessments in Section 4.2.

**Table 1:** The vision for better public health and patient safety.

**Vision**	**Description**	**Assessed in section two**
Enhanced genomic integration	PHM assess omics data, as HPO policy action predictors and intercepts	AISI evaluate BM action AIRR analyze data (4.2.2–4.2.3)
Data features that predict and intercept	PHM AI evaluate health prediction and precise care through BM classifications	AISI evaluate Gen AI quantum computing for action (4.2.3)
Enhanced physical–mental ontology	PHM track HPO classifiers for public health, patient safety and parity	AISI task and feature socio-environmental factors (4.2.3)
Enhanced NHS long-term plan	PHM monitor NHS LTP and quantum scale genomics networks with AI	AIRR cluster BM predictive health and precise care (4.2.3)
PHM of HPO with BM classification	PHM with HPO Policy monitor public health and patients' safety	AIDRS stewardship develop BM and adopt HPO (4.2.4)

**Table 2:** The goals that envision socioeconomic success.

**Goals**	**Description**	**Assessed in section two**
Community-driven data share	Public inclusiveness is a principle on data access and use in a PHM mission	AISI quantum data shares in federated learning (4.2.2)
Allocate fair resources	Resource research for equitable HPO across underserved regions	AIRR develops clusters of predictors and intercepts (4.2.3)
Workforce development	Stakeholder engagement to develop BM, authorize and adopt training on capabilities	AIRR develops AI training and skill building (4.2.3)
Public inclusiveness	Societal, UK newborn screen to Senior citizen AI genomics network	AISI evaluation of AIRR in life is Global (4.2.2–4.2.4)
Equitable digital access	PHM missions HPO Policy with BM align the previous descriptions with digital access	AISI evaluation with AIRR and AIDRS stewardship (4.2.2–4.2.4)
Economic models	PHM funding models ensure sustained progress in socioeconomic HPO-BM equity	The GDS with the AIDRS ensure finance models (4.2.3)

**Table 3:** Ecosystemic privacy and security objectives.

**Objectives**	**Description**	**Assessed in section two**
Encryption methodology	PHM of genomics and multi-omics data are safeguarded by encryption aligned to BM	AISI evaluate and action BM privacy and security (4.2.2)
Federated learning	PHM protect privacy as innovation validate HPO classifications in aligned policies	AISI evaluate and action BM federated learning (4.2.2)
Algorithm transparency	PHM deemed fair and accountable surround healthcare while shaped by transparent BM	AIRR clusters validate algorithms for fairness (4.2.3)
Governance and oversight	PHM governance ensures ethical adherence to privacy principles established for BM	AIDRS provides governance and oversight (4.2.4)
Secure data ecosystems	PHM sensitive data handling in an ecosystem drive HPO secure standard classifications	AIDRS ensures robust privacy and cybersecurity (4.2.4)

**Table 4:** Equality and parity foresight.

**HPO foresight**	**Description**	**Assessed in section two**
Standard vocabulary	HPO policy classify disease consistently in an ecosystem for improved parity	AISI develops to action BM classifiers for adoption (4.2.4)
Inclusive genomic research	HPO policy inform on genomic phases and a digital alignment of solutions	AIRR inclusive BM research in omics analysis classify (4.2.3)
AI access across regions	HPO policy set public/private partners for BM access as classifications	AIRR aims for equitable access to AI solutions (4.2.3)
Ecosystem design	HPO policy content in a PHM Mission steward equality with classifications	AISI, AIRR and AIDRS align PHM aims (4.2.2–4.2.4)
Universal HPO policy	Policies align to the AIDRS HPO policy for public inclusiveness and BM adoption	AIDRS develop HPO project for assessment (4.3.2)

#### 4.1.1 Public health and patient safety vision

Figure 1 depicts the vision of public health and patient safety in mission-sound metric evaluations with a rigorous ethical assessment of data patterns in BM for personalized HPO. Table 1 shares this vision for better public health and patient safety.

Figure 1 and Table 1 simplify HPO by stewarding data in BM as the vision for public health and patient safety. PHM infrastructure is a unified mission of which public and practitioner bodies like the Health Security Agency and Health Service Safety Investigation Board (HSSIB) are proactive in safe space with scientific capability in genomics to network BM predictors and intercept HPO as the primary care (Health Security Agency, 2025; HSSIB, 2023). In defining the HPO, genomics data knowledge interoperates to pre-empt physical and mental ill health while factoring in social and environmental sciences (Benjamin et al., 2024; Polcwiartek et al., 2024). When structured, digital BM from newborn screens action the NHS long-term plans as germline WGS insight ontology and single-cell oncology queries reform medicine with science and technology foundations (Genomics England, 2021; Palmer, 2023).

#### 4.1.2 Goals for socioeconomic success

Figure 1 showcases socioeconomic success with PHM intrinsically linked to data stewardship and ecosystem ethics through agile methodologies, which expand the morals of BM in a responsible, safe space. Table 2 describes the goals that envision this PHM socioeconomic success.

Table 2 outlines the goals for creating data–digital environments, promoting fair informatics access, and ensuring the ethical and equitable use of biological and economic models. The integrated care ecosystem's focus on socioeconomic success is supported by metrics and factors influencing population health outcomes, grounded in data governance (Samet, 2024). For example, allocating fair resources aims to distribute predictive and precision HPO equitably across underserved regions, increasing PHM productivity with economic impact (Gupta et al., 2025). Similarly, workforce development with data governance and trusted AI *via* stakeholder engagement in training future skills aligns PHM with socioeconomic success (NHS England, 2023; Future, 2025). PHM legality in the evolution of HPO phenomics requires that informatics guidance evolves with devices that open Gen AI-driven classifications for national growth (Information Commissioner's Office, 2025; GOV.UK, 2025e).

#### 4.1.3 The objective for privacy and security build trust

Figure 1 highlights privacy and security objectives that cultivate PHM trust and integrity in an HPO ecosystem with fit-for-purpose BM to predict and intercept disease. Table 3 describes ecosystemic privacy and security objectives while reinforcing public inclusiveness and stakeholder engagement in appropriate transparency.

The UK national pro-innovation approach to AI regulation is safe, secure, and robust in principles with transparent and accountable aims that are ecosystem contestable for privacy and security (Nance Rosie, 2024). HPO protection, like encryption, is a novel paradigm for future guidance, while the Data Use and Access Bill engage BM securely (Information Commissioner's Office, 2024). Pro-innovation recognizes AI infringes on the privacy of individuals and undermines human rights, while valid BM classifiers align public trust (UK.GOV Office for Artificial Intelligence, 2023). Transparent PHM accounts for all decisions, which rely on testbeds with security and protection toolkits for NHS HPO (UK.GOV Office for Artificial Intelligence, 2023; NHS England, 2019a). The National Cyber Security Centre and Cyber Security and Resilience Bill fulfill PHM data security and citizen privacy in BM, which evolves public trust and transparency across sectors (National Cyber Security Centre, 2025a; GOV.UK, 2025c; Micco et al., 2025).

#### 4.1.4 Equality and parity through scientific foresight

Figure 1 showcases equality with equal access to scientific data themes foresight as an ecosystem seeks equivalence, ensuring fairness in health and social care functions for a balance in value-based care. Table 4 describes equality and parity foresight from the HPO perspective, which culminates in policy aims prioritizing classification assessments.

PHM integrates research with equality by sharing genomic data themes and ensuring access to screen foresight for identifying at-risk individuals with pathology segmentation for interventions and mitigating adverse events (National Human Genome Research Institute., 2025). We can improve parity by analysing diverse social and environmental science data, which patterns disease classification and our impact on the planet (National Human Genome Research Institute., 2025; Chavan, 2025). Global HPO foundries steward data from groups in need to develop fair predictive models and intercepts, enhancing equitable health outcomes as data diversity grows (OBO Foundry, 2024). Universal HPO, design vocabulary, and higher expert computing foresee the BM classifier development, authorizing predictors and intercepts that adopt PHM as personal healthcare and welfare (Blobel et al., 2023).

### 4.2 Science and technology evaluation, resource, and appropriate regulation

Figure 2 cycles science and technology foundations with the AISI, AIRR, and AIDRS for continuous improvement in public health, patient safety and parity. Sections 4.2.1–4.2.4 assess an integrated care ecosystem with medical science data and AI technology founded on the BM evaluation, HPO resources, and PHM regulation. Tables 5–8 scope and assess entities for method developers, commission authorities, and adopters of classifiers. Figure 2 cycle activities for ecosystem appraisal as follows:

- Activity 1: Assess infrastructure with cooperative agile groups and pipelines, thereby ensuring a robust PHM mission with HPO policy that stewards classifications (GOV.UK, 2024a).
- Activity 2: Develop BM from upstream data as alliances cluster quality intelligence from scientific themes that feature in accurate analysis (U. K. Health Data Research Alliance, 2024).
- Activity 3: Authorize HPO to use case classifications with quantum federated learning and hybrid AI practices with privacy over accurate insights (Ren et al., 2023).
- Activity 4: Adopt PHM workstream classifications in Gen AI models, such as GPT-5, that predict health and precision care (Gil and, 2024).
- Activity 5: Evaluate infrastructure to develop, authorize and adopt BM with HPO in progressive PHM with ecosystem engineering initiatives for public safety (Carayon et al., 2020).
- Activity 6: Integrates expert public health with responsible citizens as personalized plans integrate ecosystem care for a national safety strategy (NHS England, 2019c).

**Table 5:** Critique science and technology foundations.

**AI principle**	**Science and technology foundations that mission the management of population health**
Understand AI and its limitations	PHM-enabled HPO risk stratification and disease segmentation to establish realistic BM expectations as progressive.
Lawful, ethical, and responsible AI use	PHM must comply with moral and interpretive standards while integrating equitable HPO predictive health and precision care.
Secure AI applications	PHM ecosystem data integrity needs to be federated and strengthened for multi-omics and onwards to images and social factors in HPO.
Human control at key stages	PHM maintains critical decision points in HPO that coordinate clusters and other research resources for predictor and intercept adoption.
Lifecycle management	PHM scales HPO tools consistently across BM predictors and intercepts in iterative digital twin lifecycles.
Use of the appropriate tools	PHM genomic predictive health pre-eXams and precise care eXams agile methods match expert HPO points of need in an autonomous flow.
Open, collaborative engagement	PHM promotes public inclusiveness while engaging public/private AI stakeholder partnerships on the HPO.
Collaboration with commercial teams	PHM with cross-discipline expertise for seamless application of medical use cases built from quality scientific data for safe HPO across teams.
Skill-building and expertise	PHM develops projects and competencies for HPO AI hybrid practitioners in the public/private sectors.
Align PHM mission with HPO policy	The synchronisation of PHM with HPO policy integration is multifaceted with national stewardship and international guidance in Section 3.

**Table 6:** The AI institute higher expertise in securing biological modeling.

**Science of BM disease**	**Evaluation**	**Secure and safe points of need**
Genomics BM	Advance diagnosis and predictive foresight with biomarker precision insights	Cyber-essential BM pre-eXam aids precise eXam classifiers.
Multi-omics BM	Identifies with predictors for precise biomarkers automatically	Cyber assesses eXam intercept from the personal pre-eXam classifier.
Socioenvironmental factors in BM	Explores external factors and elements that impact medical and physical health	Cyber response to HPO pre-eXam or eXam intercepts.
BM for HPO terminology	Aligns the above with or without physical imaging and mental assessments	NCSC collaborates on secure HPO classifiers.
Rare disease BM	Genome-multi-omics network predictors primarily in newborn screening studies	BM classifier consent: rare disease management.
Major condition BM	Stratifies chronic condition risk with poly-gene aetiology often factor impacted	BM classifier consent: chronic disease management.
Biopharmaceutical BM intercepts	Notify kinetics and dynamics. In addition, align BM to accelerate drug safety trials	Security assessment and consent of eXam classifier providers.
Physical BM for wellbeing	AI-driven practitioner and citizen predictors to insight fitness solutions	Guide on secure pre-eXam and eXams for citizen trust.
Mental BM for wellbeing	AI-driven personalized therapies using predictive analytics	Guide on secure pre-eXam and eXam for mental wellbeing.
Data privacy and security	Stringent patient data protection of their biological model	Secure classification protocol for citizen protection.

The HPO row in column one infers a common vocabulary and the impact of social and environmental elements, albeit BM, is a generic term. Genomics, BM or HPO pre-eXam are defined in the X, as are the intercepts.

**Table 7:** The AI research resource the classifications.

**Cluster and classify**	**Instances of AI secure applications from research to routine PHM**	**Reference resources are diverse for HPO**
UK infrastructure—A PHM ecosystem	42 ICBs, 200 PLACES, and 1250 primary care networks	Integrated care system cluster AIRR for BM and HPO
HPO Gen AI as points of need	Facilities become nuanced patient–provider communication for HPO	AISI and AIDRS with AI in health and communication studies
Quantum intelligence	Expands genomic, BM, HPO and PHM data processing capacity and speed	Research advanced by UK quantum intelligence
Federated data platforms	Secure research aligns with the PHM of HPO as the primary care	UK cloud data federations
Intelligence within security	AI use protects the PHM infrastructure from cybersecure threats and breaches	National cyber Security centre
Intelligence in informed consent	Consent protocols are simplified by AI for public/patient inclusiveness	NHS digital consent. Proposed community consents
Risk stratified predictors	Identifies high-health risk populations using scalable predictive HPO	NHS risk stratification programmes
Disease intercepts	High mortality and morbidly rates are HPO identified for early prevention	Chronic disease Prevention studies
HPO algorithm Validation	A plethora of diagnostic tools align AI accuracy and minimise bias	Alan Turing Institute research
HPO data interoperability	Integrates the ecosystem for efficient data use across platforms and sectors	Interoperability Standards

**Table 8:** AIDRS ecosystem authorization requirements for PHM classifications.

**AIDRS assessment**	**Developers responsibilities**	**Adopters responsibilities**
Transparency Interpretive Trustworthiness	Clear documentation of algorithms and methodologies to support interpretive understanding.	Focus on understandable outputs and interpretive guidance to build trust.
Scalability Ecosystemic Confidence	Development of adaptable systems that integrate seamlessly into diverse healthcare environments.	Confidence in the scalability of AI systems across various clinical workflows and settings.
Usability Equity Accessibility	Ensuring intuitive design, equitable access, and usability for all, addressing healthcare disparities	Promoting accessible adoption and bridging gaps in equity while ensuring effective use
Fairness Bias Assurance	Implementation of bias detection and mitigation strategies to ensure fairness in outcomes.	Assurance of fairness and awareness of any residual bias in AI-driven solutions.
Metrics Validation Effectiveness	Establishment of rigorous validation processes and defined metrics for accurate performance assessment.	Interpreting key performance metrics and validation procedures to evaluate effectiveness.
Track Trace Monitor	Creation of systems to monitor ongoing performance and efficiently identify emerging issues	Awareness of tracking mechanisms and leveraging monitoring systems for continuous improvements
Risk Management Safety	Integration of proactive risk management strategies and comprehensive safety protocols.	Confidence in safety measures and risk reduction processes within clinical applications.
Ethical Regulatory alignment	Adherence to ethical guidelines and compliance with national regulatory standards	Assurance of regulatory compliance and alignment with moral norms for trust in AI adoption
Data Privacy Security	Implementation of robust safeguards to protect patient data and ensure privacy.	Confidence in secure handling and storage of sensitive information within healthcare solutions

An iterative WGS-multi-omics cycle develops BM predictors and authorizes precise HPO intercepts for adoption (Bull et al., 2020; Park et al., 2024). Trusted Research Environments progress PHM in an ecosystem of BM classifications and HPO terminologies (Weise, 2024). Global integrated ecosystems share digital genomics founded in science and technology evaluations with secure and safe intelligence with research resource cooperatives and stewardship (World, 2024).

#### 4.2.1 Science and technology foundations

Figure 2 depicts key science and technology foundations in a cyclical assessment of activities for BM and HPO-driven disease prediction, precision, and prevention derived from cluster patterns identified in our digital twins (Kerr et al., 2020). Consider the following critical points, which are the bedrock of ecosystemic PHM.

1. The key scientific foundations emanating from the genomic AI network assess the common newborn rare diseases, featuring variant calls, intron intelligence and epigenetics marking to intron intelligence (Kerr et al., 2020). The scientific aspects pertinent to high-risk oncology patients include germline and single-cell origins, while major comorbid diseases exhibit polygenic risks (Ortega-Batista et al., 2025; Jain et al., 2023). HPO-tailored intercepts span multi-omics and socioenvironmental factors, as genome predictive health pre-eXams align drug dynamics and kinetics in an era of Bio eXams (Ingelman-Sundberg et al., 2023).
2. The key technical foundations emanating from the AISI, AIRR, and AIDRS assess the solutions across underserved communities to overcome digital ecosystem barriers through open science frameworks (Zarghani et al., 2024). The foundations underscore the importance of stakeholder engagement and cooperation to include the public in their gen AI ecosystem with HPO classifications annotated (Murphy et al., 2024). Therein, the practicality of informed consent and federated learning invites the public to assess data privacy and security, enabling AI transformation to process sensitive genomic datasets (NIST, 2025).

Table 5 science and technology foundations mission the management of population health, whereby UK national guidelines presented in an independent review action health and social care reform.

Table 5 outlines the scientific data themes for technological advancements that underpin modern medicine progress in the PHM mission for BM classifications with HPO terminology. Figure 2 highlights a cycle of scientific patterns in end-to-end workflows, with AISI evaluations, AIRR clusters, and the AIDRS, which drive the security, safety and use of authorized PHM in an integrated care ecosystem with continual BM improvements adopted as authorized.

#### 4.2.2 The AISI with higher experts assess BM security and privacy

Figure 2 depicts the AISI with a higher expert assessment to protect BM data, while quantum federated learning includes the public and engages stakeholders to classify ontology securely (NIST, 2025; Adach et al., 2022). The AISI will develop to provide a comprehensive and compliance approach to cybersecurity as healthcare systems using algorithms across medical science and technology transformations rely on higher expertise for secure BM (Ghongade, 2025). National regulations for privacy in the Data Uses and Access Bill, with the national cybersecurity dictum that followed, would align decentralized data access across secure genomic networks, biobanks, imaging, and life science sectors with citizen privacy preserved (UK Parliament, 2024; Department for Science, 2024). Table 6 highlights the AI Safety Institute transition to the AI Security Institute as higher experts chaperone BM.

In Table 6, the real world shows key technology with pivotal roles in an ecosystem, particularly in analysing secure genomic medical science data with privacy in personal BM interventions (Dias, 2019). Indeed, addressing the complexities and biases in large datasets requires cooperative and privacy-conscious methodologies to ensure application across populations through privacy-protected BM (Alemu et al., 2025). A solution lies in decentralized learning, as quantum capacity enables secure data (Liu et al., 2025). Ensuring secure foundations requires robust ecosystem guidance, such as those provided by national cybersecurity centers (National Cyber Security Centre, 2025b). Maintaining the confidentiality of sensitive health information within an ecosystem is paramount, mirroring established best practices for confidential arrangements (Zanke, 2024).

As detailed in Table 6, the AISI, using its expert knowledge, is key to ensuring advanced BM are robust and reliable through careful evaluation and setting of strong security rules across different science themes (AI Security Institute, 2024). This thorough expert checking aligns with other institutes, which is especially important as BM is being used more in sensitive health fields like genomic-multi-omics studies, which securely manage both rare and common diseases, each needing specific safety designs (The Alan Turing Institute, 2023). Expert assessments engage other countries, which build public trust from the secure and ethical use of BM in healthcare, protecting citizens with responsible personalized progress (Swiss Institute of Bioinformatics, 2024), as higher cybersecurity experts also assess BM privacy with the public.

#### 4.2.3 The AIRR with medical sciences cluster safe HPO transformations

Figure 2 shows a cyclical ontology process where the AIRR advances health and social care capabilities, aligning with the mission intent to scale opportunities in an action plan (GOV.UK, 2025a). A core AIRR function in Gen AI includes large language models (LLMs) and other ML/DL techniques for spatial HPO analysis while enhancing the safety and reliability of diagnosis and patient outcomes (Groza et al., 2024). Genomics Medical Sciences AI Network, Royal Medical Colleges, National Biobanks, and Life Science consortia ensure that high-quality scientific data themes process accurate HPO, accelerating knowledge extraction with graph-based representation (Buehler, 2024). As seen with the acquisition of Clinical Laboratories, Bioscience testing services present AIRR opportunities to enhance safety in precision by change HPO terms and embedding evidence or probability-based retrieval (Pathology in Practice, 2025). The AIRR supports safe HPO assessments in tech predictions of complex phenomics data that cluster patterns, contributing to ecosystem safety in broad PHM ambitions which federate secure and safe global models (Patel et al., 2022).

The X20 fold ecosystem expansion, driven by PHM-informed AI, is an action plan to enable real-time personalized analysis that supports ethical and fair practices for improved wellbeing and welfare (GOV.UK, 2025a). Genomics Medical Sciences with Royal Colleges and biobank bioinformatics for HPO apply graphical neural networks for disease prediction based on features (Zhang et al., 2021). Futures in GPT-5 enhance the AIRR's ecosystem capabilities for predictors, creating HPO-based knowledge graphs as interdisciplinary connections for safer intercepts whilst accelerating scientific theme discovery (Swiftbrief, 2025). Advanced Gen AI, particularly LLMs trained with human feedback, improves HPO annotation for building computationally tractable knowledge and ensuring safety (Ouyang et al., 2022). Transformer architectures beyond NLP into computer vision also offer advanced phenomics image analysis to enhance diagnostic accuracy and safety (Dosovitskiy et al., 2020). AISI-AIRR federated learning's role in BM “smart” HPO predictors supports privacy-conscious data analysis for secure–safe target therapy when dealing with bio-temporal data (Patharkar et al., 2024).

Table 7 highlights the AIRR that clusters predictors and intercepts through Gen AI practices in the PHM ecosystem. Table 7, row 1, describes how HPO-driven approaches within the Integrated Care Ecosystem unite 42 ICBs to 1,250 Primary Care Networks with hubs designed to place genomic capacities in a pathological HPO end-to-end workflow. Rows 2–10 of Table 7 focus on scaling AIRR for more robust HPO capabilities as higher experts in medical sciences evaluate progressive PHM. Additionally, Table 7 aligns agile methods as iterative approaches that integrate diverse data for safe HPO-driven practices, as explored in applying healthcare seamlessly (Ahmad, 2023).

Table 7 elaborates on the AIRR HPO clusters' evidence classification for a national PHM ecosystem. Hence, fairness in the integration of AI is paramount for the safe and equitable deployment of HPO-based solutions developed through, as highlighted in reviews for AI in healthcare (Ueda et al., 2023). Advancements in quantum computing amplify the AIRR's analytical depth, paving the way for innovative and safe HPO transformations as stakeholder collaboration and public engagement shape emerging metrics for enhanced PHM applications (Tomasetti, 2025).

#### 4.2.4 The AIDRS steward population health management “safely”

Figure 2 illustrates the progressive PHM hat cycle secure BM and HPO terminology for authorization. It integrates safe HPO practices and reflects how evolving ethics reshape expert medical science collaborations and algorithms to balance bias with clinical practice trade-offs in a dynamic HPO ecosystem (Hoche et al., 2025). Acting as a modern oracle, an AIDRS adapts to humane moral judgments while guiding developers and adopters on the rigorous bias, metric, and privacy authorization processes that align key regulatory bodies and ensure trust and fairness in safe AI solutions (Crockett, 2025). Four key bodies support AIDRS PHM project developers with authorization of BM adoption in the classification assessed.

1. The health research authority (HRA) safeguards the public interests in health research by emphasizing ethical practices, transparency, and public inclusiveness (NHS Health Research Authority, 2017). Meanwhile engaging in the genomics AI network classifications would authorize research biological digital modeling in primary care with four principles for public inclusiveness to inform and engage on progressive PHM (NHS Health Research Authority, 2017).
2. NICE enhances health and social care by providing testing pathways and guidance grounded in eight principles that prioritize clinical and cost-effectiveness (National Institute of Health and Care Excellence, 2025). It has further announced plans to transform the healthtech programme, aiming to embed public/private partnership engagement as a norm in the evolution of Human Phenotype Ontology-based population health approaches (National Institute of Health and Care Excellence, 2025).
3. The Medicines and Healthcare Products Regulatory Agency (MHRA) ensures the efficacy of medicines and medical devices by regulating AI integration across seven key areas, including cell and gene therapies, pharmacogenomics, and digital twins, where five principles of risk-proportionate regulation of AI support innovation and safety in BM (GOV.UK, 2024d).
4. The Care Quality Commission (CQC) ensures that health and social care services provide people with safe, effective, compassionate, high-quality care and encourages provider improvement by monitoring the quality and safety of AI-enabled services (Care Quality Commission, 2025). The CQC reviews compliance with care standards and measures patient outcomes while updating practitioners on ecosystem pangenome thinking and assessments through classifications (Chikhi et al., 2024).

In AIDRS alignment, the HRA and MHRA deployed new UK clinical trial regulations embedding combined reviews into law (GOV.UK, 2025b). Classification authorization and BM adoption support NICE, which embraces the responsibility of genomic science and technology assessments that potentiate HPO clinical pathways (NICE, 2025). Oversight from the CQC, directed by the DHSC, may further align with the NICE goals to appropriately structure an AIDRS for BM classifications to support future HPO stewardship with appropriate regulation (Care Quality Commission, 2021; GOV.UK, 2024f).

Figure 1 aligns the genomic AI network and the AIDRS, which are pivotal for a PHM ecosystem expansion that authorizes BM classifications and stewards HPO terminology in a global harmonization. Indeed, Table 8 depicts AIDRS authorization requirements.

Table 8 details the authorization requirements for developers and the arrangement or assurance placed on adopters within the AIDRS ecosystem concerning PHM classifications. Key considerations include transparency, scalability, usability, fairness, metrics, tracking, risk management, ethical alignment, and data privacy, which stakeholders address to ensure the responsible and effective implementation of AI in PHM.

A. The stewardship of AIDRS for developers involves guiding the BM commission of pathways for classical AI-driven predictors and intercepts for medical reform, outlining protocols in a national assessment mechanism to ensure that PHM aligns with national goals and norms in an ethical and safe progression of innovations for clinical application (National Institute of Health and Care Excellence, 2025).
B. The proposed authorization of AIDRS classification necessitates the rigorous evaluation of algorithms and methods, focusing on interpretability, validation against defined metrics, and fairness monitoring for effectiveness and safety (NHS Health Research Authority, 2017; National Institute of Health and Care Excellence, 2025; GOV.UK, 2024d; Care Quality Commission, 2025; GOV.UK, 2025b; NICE, 2025; Care Quality Commission, 2021; GOV.UK, 2024f; National Institute of Health and Care Excellence, 2025). This process ensures that AI-driven classifications meet established standards before deployment in PHM, with trust, reliability and cost-efficient applications (NHS Health Research Authority, 2017; National Institute of Health and Care Excellence, 2025; GOV.UK, 2024d; Care Quality Commission, 2025; GOV.UK, 2025b; NICE, 2025; Care Quality Commission, 2021; GOV.UK, 2024f; National Institute of Health and Care Excellence, 2025).
C. The stewardship of AIDRS for adopters focuses on building PHM trust as effective technologies with rigorous metrics and cost scrutiny authorize classifiers that adhere to standards (NHS Health Research Authority, 2017; National Institute of Health and Care Excellence, 2025; GOV.UK, 2024d; Care Quality Commission, 2025; GOV.UK, 2025b; NICE, 2025; Care Quality Commission, 2021; GOV.UK, 2024f; National Institute of Health and Care Excellence, 2025). Assurances for seamless adoption of classifications integrate regulated projects as higher expert medical sciences use safe tools that drive public trust in classified AI-driven predictor and intercept solutions (National Institute of Health and Care Excellence, 2025; GOV.UK, 2024d).

### 4.3 The ecosystem: PHM mission, HPO policy, and HEMSS classifications

Figure 1 expands the UK DHSC and DSIT strategies in a PHM Mission as an AIDRS-genomics AI network policy for HEMSS to navigate classical clinical decision-making (National Health Service, 2024b; Hassan et al., 2023). World Health PHM and United Nations Sustainable Development Goals for wellbeing and economic growth are accelerated (The World Health Organization, 2023; United Nations, 2023; The Global Goals, 2023) as this HPO ecosystem broadens (Gargano et al., 2023).

Figure 3 mission one national policy to steward development, authorization and adoption of classifications as actioned in Sections 4.3.1.–4.3.4. Tables 9–12 mission PHM, plan HPO policy, action mature experts' genomic medical sciences to predict health in pre-eXams and precision care through eXams [X=Gen AI].

**Table 9:** The population health management five point mission.

**Mission point (scientific data)**	**PHM mission description**	**Key ecosystem actions**	**National expected outcomes**
Genomics AI network	Develop research ops for authorization	Data spoke for BM points of need	Personalized genomics pre-eXams for eXams
National image Cooperatives	Anatomical to nucleotide data scans	Data spoke for HPO value-based care	Personalized HPO norms monitored by imaging
Socioenvironmental determinants	General practice and welfare informatics	Data spoke for HPO and BM parity	Address access to health determinants and educate
Data Biobanks align hybrid AI	Ecosystem development of real-world predictors	BM and HPO norms align predictors	Integrate HPO research as valid classifiers
Life science aligns hybrid AI practice	Life science—bio- and multi-omics intercepts	PHM of HPO align predictor to intercept	Authorize eXam intercept from valid BM pre-eXam

**Table 10:** AIDRS HPO policy align entities with HEMSS actions, impacts, and outcomes.

**AIDRS entities**	**HPO policy stewarding actions**	**HPO policy impact and outcome**
Propose AIDRS HEMSS	Implement HEMSS and adopt BM classifiers and HPO vocabulary in a digital standard approach to PHM goals with agile methods scaled.	Steward classification authorization ensuring BM supports PHM initiatives for personalized citizen wellbeing and healthcare equity.
Public Inclusive	Facilitate a lifetime journey in a healthcare digital twin explanation with metrics to support the point of need to steward safe, secure PHM	Expectations of healthcare AI and the role of trust require understanding views on how AI will impact health access and patient–provider relationships
GDS GMS	Facilitate genomics AI network (GMS/GDS) *via* PHM stakeholders with an ethical BM integration that eco-systematically align global HPO	Ensures HPO policy development with robust data science themes and agile methods in an effective use of Gen AI for scaling accurate BM classification.
HRA MHRA NICE	Authorize research protocols with ethical compliance. Regulate AI use in BM devices and verify classifiers from AI-genomics assessments.	Drive responsible PHM while adhering to ethical and safety standards with secure integration of AI tools for BM improvements in wellbeing.
AISI AIRR	Facilitate AISI evaluations for HPO-linked datasets; Provide guidance on AIRR protocols for safe PHM AI model development and validation.	Enhance trust in the secure handling of sensitive phenomics data promotes the development of safe and reliable AI models for PHM applications.
ICS	Implement genome science and AI tech with PHM stewarding of HPO and BM aligning quantum federated learning in a medical Gen AI reform.	Accelerates healthcare innovation and equips ecosystem skills to adopt and manage classical HPO as ecosystem capacity and capability expand.
CQC	Conducts performance assessments of ICSs to evaluate and enhance value-based care in the PHM of BM with tools, norms and guides.	Maintains patient safety, enhances public health, and ensures parity through tech monitoring of BM toward a digital HPO ecosystem.

**Table 11:** HIMSS maturity for developments and HEMSS adoption.

**HIMSS group maturity Advisory levels: classifiers develop**	**HEMSS principles Stewardship: classifier adoption**
HIMSS levels 0–7 provide a technical infrastructure framework for project development, requiring strong stewardship for effective PHM integration.	HEMSS aligns GDS and GMS strategies in a PHM mission within a hybrid cloud environment, with public inclusiveness and stakeholders' engagement in BM and HPO classifications.
IFRAM assesses IT integration across ICB regions for HPO transformation and value-based BM care through its Infrastructure Readiness and Adoption Model.	EXPERT alignment of cross-discipline hybrid AI practitioners, which benchmark PHM evaluations for classifier authorization through XAI metrics and fairness.
AMRAM evaluates AI integration in clinical workflows, enhancing agile methodologies and anatomical oversight through its Adoption Model for Application of AI in Medical and Radiological Management.	MEDICAL includes pharma, life science, and biobanks with genomics public–private partnerships for safe and effective HPO adoption, with AIDRS/CQC assessment in trusted AIRR environments.
EMRAM provides essential data sources for AI analysis, supporting HPO classification and predictive health through Electronic Medical Record Adoption Models. Albeit the future migrate genomics' personal data storage	SCIENCE uses data themes from multi-omics, imaging, and health determinant data for digital twins, with HEMSS stewarding the adoption of AIDRS classifications across the integrated care ecosystem.
CCOM facilitates information sharing for longitudinal patient tracking, requiring national stewardship for project cohesion through the Continuity of Care Record Adoption Model.	SAFETY ensures public health, patient safety, and parity *via* secure data handling and citizen HPO access, including pre-eXams and eXams classifications.

**Table 12:** Genomic AI network predictive health pre-eXam with precision care eXam.

**The pre-eXam Predictor**	**The eXam Intercept**	**X is fair, transparent BM in HPO vocabulary**
Biological genomics predictive health modeling	Biological disease intercepts for model wellbeing	Fair and transparent biological models
HPO standard predictive health modeling	HPO disease intercepts for trait wellbeing	Fair and transparent HPO standards
Algorithmic bias detection and mitigation	Personalized care plans with transparent reasoning	Algorithmic fairness (implicit/explicit)
WGS germline, single cell or tumor assessment	Culturally sensitive communication and care delivery	Equity in interpretation and public inclusion
Socioeconomic vulnerability screening	Resource allocation based on social determinants of health	Social fairness with Community inclusion
Geospatial, trend, and image screening	Location-based on specific interventions on escalations	Interpretive accuracy for actions

Genomics AI develops BM predictors for intercepts and is authorized for use in pre-eXam and eXam classifiers. X is transparent on metrics and bias mitigation as is practicable, while x is in a pre-exam or exam developed to attain authorization through X due diligence in fit-for-purpose and cost-effective classifications.

#### 4.3.1 Population health management, a five-point mission

From Figure 3, The PHM five-point mission commences genomic scientific data alignment with multi-haplotype pangenome graphs as quantum-Gen AI scale BM and reform healthcare in HPO patterns (Secomandi et al., 2025; Flöther, 2023). The global genomics building blocks of life are the PHM mission enabler for the AIDRS (AISI security, AIRR safety) to develop, authorize, and adopt classifiers in a standard vocabulary (Murphy et al., 2024). Table 9 mission PHM in five points as a roadmap for the BM of scientific data themes in primary HPO care.

Table 9 shows that key ecosystem actions and national expected outcomes progress with AIDRS stewardship of PHM to improve the quality of health life expectancies while mitigating care inequalities. Table 9 mission points with descriptions that prioritize disease prevention over treatment, aligning higher experts in medicine and sciences to develop, authorize and adopt secure (AISI) and safe (AIRR) technologies in a five-point mission as follows:

1. To advance the genomics AI network central to the PHM mission, the DHSC engages in developing an international strategy for genomic data, setting robust standards for its collection, secure sharing, and ethical use (Lee et al., 2025). A PHM ecosystem classifies clinically relevant genomic variants and develops robust predictive models with well-defined genomic-based diagnostic and prognostic tools for HPO with the royal colleges and genomic network (Lee et al., 2025).
2. To realize the potential of imaging from anatomical to nucleotide-level data within PHM, NICE, guided by input from AIDRS, assesses standard acquisition methods and precise annotation of medical imaging using HPO terminology (UK Health Data Research Alliance, 2025). Enhancing capabilities in this domain requires strategic investment in developing and deploying AI-powered imaging analysis platforms that can integrate with HPO to improve early detection and monitoring across ICSs (UK Health Data Research Alliance, 2025).
3. Addressing global PHM with social factors requires coordinated action from multiple bodies such as the UK HSA and NICE to implement a standard in systematic data collection, linking information to HPO profiles across health and social care (World Health Organisation, 2025). To use data effectively, develop and evaluate target community intercepts to address health inequalities and improve literacy, carefully considering ethical implications, fairness and reform metrics (World Health Organisation, 2025).
4. Effective use of bio-banks for PHM relies on ICSs enhancing interoperability and establishing clear, accessible protocols for researchers focused on HPO-linked BM development, spanning the genome to the proteome (UK Biobank, 2025). The utility of these resources strongly recommends the comprehensive integration of diverse omics datasets with detailed, accurately annotated structures under established data stewardship that idealizes real-world studies (UK Biobank, 2025).
5. Translating life sciences research into impactful multi-omics intercepts for PHM requires establishing a transparent, adaptive regulatory ecosystem to authorize AI-driven digital twin BM interventions (BIA, 2024). In accelerating just partnerships, the UK aims to develop efficient translation of novel bio-preventative strategies with accurate intercepts identified through phenomics data, with an authorization process pivotal to assured metrics and public trust in the BM classification recommended (BIA, 2024).

#### 4.3.2 AIDRS HPO policy steward higher expert medical sciences safety

Figure 1 illustrates the AIDRS HPO policy roadmap for HEMSS to develop, authorize, and adopt primary care classifications that personalize proactive prevention to negate the “error-prone aspects of healthcare” (Antony, 2024). Scientific data themes and accessible BM tech create a “niche” of spatial multi-omics, which mitigate risk by “constructing and personalising population pangenome graphs” (Wang et al., 2024; Chikhi et al., 2024). AIDRS HPO policy stewards HEMSS for BM risk stratification in disease prediction with intercepts as the phenotypical norm portal to population biobanks for the best citizen outcomes (Bioportal, 2025; Lin et al., 2024).

Figure 3 triangulates government, independent, medical, science and technology bodies with the AIDRS [HRA, MHRA, NICE, CQC], AISI, and AIRR advancing PHM. The AIDRS, along with NICE and the Genomic Network, implements an HPO policy for agile group BM development with socioeconomic betterments, adopting classifications (NHS, 2025a). HPO policy for HEMSS stewards rigorous metrics in AI comparatives as fair BM spans research to authorize ecosystem integration of classifiers at the point of need (The Association of Clinical Biochemistry and Laboratory Medicine, 2023). Table 10 depicts the AIDRS HPO policy, which aligns entities with HEMSS actions for impacts and outcomes.

Table 10 shows the genomics-AIDRS HPO policy stewards' developers and adopters in authorizing predictors and intercepts that consolidate principles from key entities in the progressive PHM of BM. This builds trust and clarifies developer expectations of healthcare AI and user adoption while engaging stakeholders and public inclusiveness upon progressive PHM in HPO policy (Nong, 2025). From Table 10, the genomics AI network aligns an AIDRS HEMSS resource to adopt quantum federated learning with Gen AI evidence for public perspectives on HPO transformation (NHS Genomic AI Network, 2025; Genomic AI Network, 2024). The genomics AI network scale BM solutions in classifications as HEMSS evaluate metrics with fairness in the PHM of HPO progressing more complex points of need in a cycle of continual improvement (Genomic AI Network, 2025).

#### 4.3.3 Higher expert medical science safety stewards classifications

Figure 3 depicts a national integrated care ecosystem in which an AIDRS-NICE-Genomics Network commission PHM progress with BM classifiers proposed that evolve standard HPO. A National Health Service HIMSS partly guides BM progression levels (Burrell, 2023). While HIMSS interoperates for the HPO transformation, limitations with maturity and benchmarking remain for a collective PHM mission (News Editor., 2023). These include as follows: -

1. HIMSS levels are a voluntary service with national science or technology oversight developing whilst an NHS struggles to maximize the PHM of BM as a primary care, whereby enhanced capacity and staged HPO capabilities require benchmarking and stewardship (News Editor., 2023; Bridges et al., 2025).
2. Specialized hospitals lack a comprehensive ecosystem roadmap in HIMSS whereby an optimal PHM mission for BM is a progressive adoption mission of assessments and trials with both AI technical guidelines and HPO policy stewarding evaluations and classifications (Bridges et al., 2025; You et al., 2025).
3. Genomics AI network research and routine services use GLIMS and HIMSS, whilst there is a need to address national cybersecurity, privacy, informed consent and the appropriateness to regulate an ecosystem on secured and trusted stewardship of the agreed classifications (You et al., 2025; Khan et al., 2024).
4. Biopharmaceuticals lack individual specificity in generic products and lack a national standard in secure genomics digital twin approaches through which an AIDRS and genomic AI network engineer adoption for a trusted ecosystem to authorize the classification in the assignment of ownership (Khan et al., 2024; Ringeval et al., 2024).

These detailed challenges are addressed in Table 11, highlighting HIMSS maturity with HEMSS principles that steward solutions for an integrated care ecosystem driven by the development and adoption of authorized predictors and intercepts.

From Table 11, Column 1, the HIMSS mature technical infrastructure that aims to develop analogue to digital projects idealize disease-to-prevention in a health and social care ecosystem (Health, 2025), which needs HEMSS stewards (Burrell, 2023; News Editor., 2023; Bridges et al., 2025; You et al., 2025; Khan et al., 2024; Ringeval et al., 2024). When authorized in classifications parts, an integrated care ecosystem personalizes each BM at each point of need as genomics-AI clusters predictors and intercepts in a PHM mission that continually renews developments (Minkman et al., 2025).

From Table 11, Column 2, HEMSS renews PHM in adopting BM classifications that authorities deem fit for purpose, whereby AIDRS HPO policy reviews HIMSS and the genomics AI network metric comparatives for performance measurements and authorization of classifiers (Minkman et al., 2025; Health Service Research, 2022). AIDRS [HRA/MHRA/NICE] principals for classic authorization use the AISI and AIRR to steward secure and safe end-to-end workflow, as HEMSS align policies to sustain good health and wellbeing (IISD, 2025).

From Table 11, HIMSS-HEMSS stewards PHM agile method developers with BM classification are authorized for the seamless adoption of ethical, safe, secure, and cost-effective predictors and intercepts. HEMSS steward's ecosystem health and safety while overcoming differences in professional opinion as a PHM mission aligns entities in standard metric classifications that simplify “multi-level health determinants” and drive competitive biopharma markets from pre-eXam predictor to eXam intercept (Koumpis et al., 2025).

From Table 11, HIMSS-HEMSS stewards national privacy with safe and secure federated learning with rigorous BM training, HPO aggregation, and global updates (Collins, 2025). PHM metrics with bias mitigation evolve explainable AI as methods personalize data-driven healthcare for a BM learning cycle that classifies the research for use (Yara, 2024; Sadeghi et al., 2024). Tracking genomics through the digital twin journey presents citizens and government with continuous PHM improvements in wellbeing and welfare (Katsoulakis et al., 2024).

#### 4.3.4 Medical predictive health and precise care classifications

Figure 3 aligns medical reform with scientific data themes and quantum-Gen AI technology with predictive health pre-eXams and precise care eXam classifications that enhance public health and patient safety, initially through genomics (Henrique et al., 2025; Khoury, 2021). PHM progress in agile methods for predictive pathogenicity continues the precision medicine transformation that developed post-pandemic (Henrique et al., 2025; Khoury, 2021). HPO policy will model biology with biobanks and life sciences when the AIDRS-NICE-Genomics Network stewards the development, authorization and adoption of classifications, as detailed in Sections 4.3.4.1–4.3.4.3.

##### 4.3.4.1 Development of genomics AI network predictors and intercepts

Figure 2 highlights how the genomic-AIDRS network aligns evolving strategies and principals, such as the alignment and processing of HPO terms, as agreed, within a secure and safe BM ecosystem. Developing predictors and intercepts projects the clinical and cost-benefit analysis, which considers privacy preservation in Gen AI applications to continuously improve the PHM ecosystem (Swetha et al., 2025). Federated data-sharing with or under the TREs incorporates an architecture that facilitates secure connections with efficient, sensitive data research processes across the ecosystem while safeguarding individual privacy in collaboration (Igbo, 2024). The genomics AI network services PHM in NHS long-term plans that work the BM ecosystem as stakeholders secure communities in classifications as hub-directories embed multi-omics with privacy in health and social care (N England, 2024).

In an international overview of genomics AI, the future and impact of organizational principles must align BM whilst enabling a nation's role of authorizing their assessments in their choice of predictors and intercepts as the foundation for personalized healthcare integration and PHM progress (Maqsood et al., 2024; Ozcelik et al., 2024). Global genomics and continental molecular initiatives that steward public inclusiveness and stakeholder engagement ensure BM access and use as data evolve moral BM in a transparent ecosystem (World Health Organization, 2024; Hatch, 2025). Developing HPO policies empowers the commission of classifications for equitable access to rare and major condition predisposition with a choice of health predictor insight that option intercepts, which builds PHM trust (Basnayake Ralalage et al., 2024). An HPO standard in genomic AI equity foresees access to predictors with secure data which advance precise target intercepts, prioritizing patient safety through future regulation that stewards innovation for digital twins (GOV.UK, 2024d).

##### 4.3.4.2 Authorize genomic predictive health pre-eXam and precise care eXam

In Figure 1, the genomics-AI digital service securely institutionalizes safe research resources that develop, authorize and adopt a PHM mission with an HPO policy to steward Gen AI. Table 12 shows and expands the genomic AI network for predictive health pre-eXam with precise care eXam intercepts, authorized as Gen AI [X] classifications for use.

From Table 12, the WGS pre-eXam shapes our future to authorize genomic BM health predictors, which refine value-based personalized care (PWC Network, 2024). Genomic-AI platforms such as MARVEL and Congenica enhance the precision of variant ranking, enabling the accurate identification of pathological variants (Harvard Medical School, 2017; Congenica Ltd, 2022). DNABert facilitates understanding of complex genomics, while DeepVariant employs neural network architectures for highly accurate NGS analysis, with actionable predictive health insight (Ji et al., 2021; Telenti et al., 2018).

From Table 12, the eXam intercepts multi-omics precisely across HPO points of need. Platforms like SOPHiA DDM and Franklin with XAI enhance genetic data interpretation with value-based care delivered as personalized biopharma, nutrition, and welfare intercepts (Alkhanbouli et al., 2025; Allen, 2024). Platforms in Galactic AI and Geneyx integrate medical science data which link genetic variants and patient characteristics as a pre-eXam couples an eXam drug target when HEMSS steward's genomics AI excellence (Taylor et al., 2022; Ocean Genomics, 2023).

From Table 12, the pre-eXam and eXam stewarding of UK Biobanks is exampled with DNA Nexus and Myriad Genetics as TRE underpins HPO primary care and BM reform. DNA Nexus, a cloud-based bioinformatics platform, underscores the importance of large-scale WGS data in refining value-based personalized care in genomic health predictors (DNA Nexus, 2025). Meanwhile, Myriad Genetics paves the way for advanced scientific data analysis and accelerated research intercepts in BM with precision medicine (DNA Nexus, 2025).

From Table 12, X is fair with transparent BM aligning an HPO vocabulary. Genomics pre-eXam Gen AI-GPT 5 classify health predictions with federated BM learning for accurate and private authorized eXams (Quazi et al., 2024). Quantum-genomics AI accelerates classifications across large-scale variants as the predictive disease is segmented and aligned with precise therapies (Ali, 2023). Seamless aggregation of multi-scientific themes necessitates ownership, as HEMSS steward classification cooperation as quantum intelligence reforms medicine (Chow, 2024).

##### 4.3.4.3 Adoption of classifications in a PHM ecosystem

From Figure 3 and Table 12, the DHSC and DSIT would develop PHM for BM adoption in a classification ecosystem which steers the TRE predictors or intercepts, authorized as fair with metrics for value-based care. Achieving large-scale seamless BM adoption of classifiers minimizes PCN variation as genomics AI align systems and principals for decision-making (Scott et al., 2024). A decade from conception, a robust PHM mitigates practice variability with a robust PHM ecosystem for BM adoption, which records deferred classifier use with science, technology, and clinical review for continual improvement (Villa et al., 2014). AIDRS HEMSS endorsement stewards' continuous improvements, which align TREs for medical reform in classifier adoption with Algorithmic Transparency Recording Standards, an option (Welsh Government, 2024). Federated learning and quantum classification with genomic AI digital twin predictors will network high-speed intercepts for adoption (Saeed et al., 2024).

PHM is the adoption of classifiers in an ecosystem that aligns patterns, commencing with DNA that develops populace disease niches and stewards HPO in a genomic AI network (Jackson, 2025; Goethem et al., 2025). The basics in developing classifications are built from digital technology assessment criteria and quality systems (Wu et al., 2025; AI, 2025). Whereas in PHM, genomics generate predictive health pre-eXams in real-time data analysis and builds beyond the central dogma summary for phenome anomaly detection, wherein multi-omics and health determinant factors are assessed for national adoption depending on their classification requirements and commission arrangements (NHS, 2025b). Refining biobank scientific themes while annotating genomics and training Gen AI on BM characteristics adopt precise eXam intercepts that personalize care (Lu, 2024; Ghebrehiwet et al., 2024). Recognizing that the life sciences industry reduces drug developer timelines, it accelerates ideals to mitigate regulatory barriers to a safe PHM programme for BM success, streamlining clinical trials to target patient care (Pharmaphorum, 2025).

### 4.4 Discussions on global medical reform for public health and patient safety

The manuscript navigates global medical reform for public health and patient safety, wherein PHM benefits from phenotype ontology policy that steward's genomics AI to model our biology in an ecosystem. The author discusses HEMSS to steward developers, authorities, and adopters of predictive health pre-eXam and precise care eXam intercepts that personalize healthcare. Hence, national science and technology foundations re-evaluate medicine for reform as the author missions PHM with national HPO policy as HEMSS steward classifier channels as follows: -

Section 4.4.1 National ecosystem safe space expansion

Section 4.4.2 National ecosystem continual improvement

Section 4.4.3 National ecosystem development, authorization, and adoption

Section 4.4.4 International population health management.

#### 4.4.1 National ecosystem safe space expansion

Figure 1 shows the ecosystem safe space with interconnected components under the DHSC and DSIT, wherein the author would “Culture Intelligent Workflow Structure and Steps (Henry, 2023)”, which cultivate genomic AI classifications to manage populace health. Expanding the safe space presents arguments for greater cooperation to accelerate PHM innovation through secure data sharing, enabling the development and validation of HPO-driven applications in a controlled environment. Conversely, arguments against expansion involve concerns around increased complexity in governance, challenges in scaling infrastructure while maintaining security, and the risk of siloed innovation, potentially hindering broader adoption across a national ecosystem.

The debate surrounding expanding national ecosystem safe spaces for PHM centers on the balance between using innovation and managing challenges. Proponents of trusted data and scientific themes integrate quantum-Gen AI and cybersecurity, leading to more effective predictive health models and personalized care pathways (U. K. Health Data Research Alliance, 2024; Weise, 2024; Dias, 2019). Cohorts may argue that a well-stewarded safe space can mitigate data privacy and security risks, thereby sustaining trust among stakeholders and the public in the NHS (Kerasidou, 2023). However, critics raise concerns about the resources to establish and maintain a safe space, questioning whether the infrastructure benefits outweigh the costs (GOV.UK, 2024a). Discussions around the potential for such spaces inadvertently create barriers, albeit ensuring equitable access and participation is a national priority based on genomic research validation of phenotype (National Human Genome Research Institute., 2025).

The author and reader need to re-examine Table 1, which outlines a foundation vision for public health and patient safety, with Table 7, which details the AIRR's role in clustering safe AI applications as a controlled and ethical PHM environment. However, the challenge arises when considering applying this model across the health and social care sector. The tables do not account for the fact that regions within the nation will have varying levels of digital readiness. A key consideration for national expansion is whether safe and ethical standards can be implemented efficiently in areas with less developed digital capabilities. The author believes Tables 1 and 7 highlight the vision and evaluation in a PHM ecosystem while classifiers also deliver on Tables 2–4 goals as science and technology foundations steward Safe Space.

#### 4.4.2 National ecosystem continual improvement

Figure 2 shows the end-to-end workflow for secure and safe BM through a cyclical process stewarded by HEMSS and guided by equitable and fair practice principles, which align with the PHM of HPO transformation (Henry, 2025d). The ecosystem is advantaged by continual improvement, with the ability to adapt to emerging scientific evidence and integrate technological advancement, refining classifications for greater accuracy and clinical utility (Henry, 2025b). Disadvantages involve the costs and complexities associated with continuous updates, disruption to established workflows, and the need for robust ecosystem processes to ensure HPO transformation enhances rather than obstructs patient care and ecosystem efficiency (Henry, 2025b).

Discussion around the continual improvement of the national PHM ecosystem, particularly concerning HPO transformation, highlights the tension between progress and stability. Advocates emphasize the necessity of adapting to the rapidly evolving fields of multi-omics and AI to network predictive health from gene interactions (Bull et al., 2020; Kerr et al., 2020; Alemu et al., 2025). They argue that continuous refinement of HPO ensures its relevance and accuracy in capturing and annotating complex human pathology traits around the world (Gargano et al., 2023; Murphy et al., 2024). However, continual improvement can be resource intensive, requiring ongoing investment in research, technology, and training in agile methods (Ahmad, 2023). The reliability of the ecosystem during periods of transformation and the need for standardized validation metrics pose hurdles which are resolved through continual improvements in classification benchmarks (Bridges et al., 2025).

Table 2 outlines goals for socioeconomic success assessed through ecosystem development arrangements and adoption requirements, whilst Table 8 details AIDRS ecosystem authorization requirements for PHM classifications, which embed a means for national evaluation in the ecosystem. These tables suggest that continual improvement is linked to achieving desired outcomes and maintaining trust. However, the practicalities of measuring the impact of continuous changes and ensuring that they are uniformly beneficial across diverse patient populations and healthcare settings require careful metrics, ethical considerations, and stewardship.

#### 4.4.3 National ecosystem development, authorization, and adoption

Figure 3 shows the triangulation of medical reform with scientific data and quantum-Gen AI classifications within an ecosystem mission, policy, and stewardship. BM resonates with the PHM of Genomic Newborn Screens with multi-omics intercepts, which focuses on the benefits of a structured national approach to develop, authorize, and adopt safety and efficacy through the evaluation of standard tools with equitable access (Henry, 2025c). Limitations include bureaucracy that slows innovation, balancing regulatory oversight with agility, and potential resistance to adopting new technologies, whilst the benefits of digital twin cycles from birth offer a lifetime of predictors and intercepts (Henry, 2025b).

The discussion around a national approach to developing, authorizing, and adopting PHM tools and BM classifications centers on the trade-offs between control and agility. MHRA-coordinated approaches to regulating devices and AI offer significant advantages in quality control and patient safety (GOV.UK, 2025e, 2024d). Other standard authorization processes involving bodies like AIDRS-NICE ensure that only safe and effective innovations are deployed at scale (NICE, 2025). Critics point to the potential for lengthy approval processes that stifle innovation and delay the adoption of beneficial technologies while BM integrates at scale (NICE, 2025). The need for continuous agile methods with unbiased training and expert comparative metrics in healthcare and medicine requires greater AIDRS cooperation in endorsing the authorization of classifications (Welsh Government, 2024).

Table 3 outlines objectives for privacy and security, aligning the PHM five-point mission in Table 9, which details scientific data themes that impact phenomics for authorized use in a multifaceted controlled ecosystem. These tables suggest that secure and safe BM risk stratification needs responsible innovation, in which primary and social care benefit from national assessments and authorizations. The challenges of seamless HPO adoption across different healthcare settings and the potential for regional variations in implementation mean that the CQC-AIDRS-AIRR–AISI-HEMSS needs to risk manage ICS assessments for capacity and capabilities. The ideal of an integrated care ecosystem to develop, authorize, and adopt classifiers is for regions to scale up and out on classifications.

#### 4.4.4 International population health management

Internationally, PHM strategies vary significantly from impact ability gaps to risk stratification, with differences in public and private health systems, cultural and ethical priorities, and socioeconomic factors (Orlowski et al., 2021). Figure 1 showcases the UK PHM ecosystem to expand international borders and contexts, as Figure 2 depicts continual improvement in AI analytics, comparatives, metrics, fairness, and monitoring. Figure 3 triangulates medical reform with quality scientific data themes and quantum-Gen AI, whilst nations unite in PHM while maintaining their classifications for authorization.

An international PHM mission for HPO adopts BM classifiers with trust in value-based care as nations align proof of concept, cost–benefit, and business cases to develop, authorize, and adopt classifications. This programme stewards an ecosystem with the Genomics and Life Science networks as public–private partners engage in an AI adoption mission. Therin the UK Long-Term Plan is accelerated by addressing HPO variability with research, assessment, commission, and stewardship of classifications (NHS England, 2019b). The UK DSIT may accelerate their global influence lead to predictive and precision medicine, with classifiers recommended internationally (Department of Science Innovation Technology, 2024).

##### 4.4.4.1 **Develop population health management ecosystems**

Developing a robust national PHM ecosystem with fit life cycles in future analytics is a mission to coordinate healthcare delivery, engage stakeholder cooperation, and promote data-driven decision-making (Henry, 2025b). Global ecosystems facilitate the integration of data from diverse sources, with the implementation of evidence-based interventions and the ongoing monitoring and evaluation of program effectiveness, which benefit from fit-for-purpose programmes like HPO Monarch and HEMSS reform (Human Phenotype Ontology, 2024; Henry, 2025a).

A strong foundation for ecosystem cluster genomic patterns evaluates populace wellbeing, presenting metrics to global bodies, as Table 4 analyses omics health features alongside environmental and social factors for parity in primary care phenotype (Samet, 2024; The World Health Organization, 2023). The critical evaluation of the UK's science and technology foundation in Table 5 underscores the need for a strategic approach to genomics and other determinants of HPO (Benjamin et al., 2024; World Health Organisation, 2025). The development of an ecosystem benefits from national HPO policy to steward genomic information into medical practice classification, aligning data presented in the tables and highlighting BM approaches to phenotypes worldwide (OBO Foundry, 2024; Gargano et al., 2023). Harmonizing open science predictors and intercepts across countries with data sharing realize rapid ecosystemic health (Blobel et al., 2023; Zarghani et al., 2024).

##### 4.4.4.2 **Authorize human phenotype ontology computing**

An HPO provides a standard vocabulary with risk stratification to predict and segment disease, which is essential in applying WGS computing technologies for wellbeing and economic growth (Henry, 2025a; Pathology in Practice, 2025). Authorizing HPO computing actions establish world-wide ecosystems for the secure and ethical use of clustered data, promoting interoperability among different systems and ensuring that tools are validated and approved for use (Henry and Pathology in Practice, 2025; Henry, 2025a). The HEMSS authorization process is critical for facilitating the development of AI-powered predictor and intercept tools, improving the accuracy of variant interpretation, and enabling precise and personalized approaches (Henry and Pathology in Practice, 2025; Henry, 2025a).

The Genomic Network architecture, as outlined in BM in Table 6, is a primary science and technology data source that intersects with the regulatory functions of the AI and Digital Regulation Service, detailed in Table 10 with action, impacts, and outcomes (National Health Service, 2024a; NHS Genomic AI Network, 2025). Other nations can facilitate medical reform by implementing authorized HPO computing that seamlessly integrates genomics into personalized medicine at their discretion (Swiss Institute of Bioinformatics, 2024; Yara, 2024), while stewarding the safe and effective deployment of trusted AI technologies (Micco et al., 2025; Khan et al., 2024). An international focus on these technology advancements, coupled with responsible HPO implementation, critically depends on establishing secure infrastructure to support the development and authorization of digital health classifications (Adach et al., 2022; National Cyber Security Centre, 2025b).

##### 4.4.4.3 **Adopt biological modeling classifications**

Global reform through BM research is crucial in advancing root cause understanding of disease predictors for accurate interceptions in the PHM of HPO (Henry and Pathology in Practice, 2025; Henry, 2025a). Standard BM classifiers ensure research evidence's reproducibility, comparability, and interoperability with HEMSS (Henry and Pathology in Practice, 2025; Henry, 2025a). This develops and promotes common standard model classification validation techniques which adopt the genomic predictive and diagnostic pre-eXam with the precise eXams [X=Gen AI], leading to improved public health, patient safety, and parity (Henry and Pathology in Practice, 2025; Henry, 2025a).

HIMSS maturity, as presented in Table 11, offers a voluntary assessment to adopt digital health solutions, while digital twins, in Table 12, complement PHM (HIMSS, 2021; Ringeval et al., 2024). Table 11 also indicates that HEMSS is crucial in stewarding standard classifier adoption of accurate, personalized healthcare, as classifiers are re-evaluated with a new pangenome (Huang et al., 2024; N England, 2024). By authorizing digital twin pre-eXams and eXams as an efficiency-driven approach to wellbeing, quantum federated learning further enhances adoption by maintaining trust through BM privacy (Collins, 2025; Saeed et al., 2024). Ultimately, global reform can realize the United Nations SDGs by adopting BM classifications that optimize health and social care for socioeconomic success (United Nations, 2023; Gupta et al., 2024).

## 5 Conclusion

This manuscript charts a comprehensive course for the future of UK PHM, driven by genomic and multi-omics advancements alongside artificial intelligence in bio-intercepts, all within a stewarded ethical and regulatory structure. It meticulously outlines the present state, emphasizing the crucial roles of national initiatives like the genomics AI network and AIDRS in building a foundation for progressive reform. Examining the HPO as a vital tool for standard data graphs and enabling complex BM underscores its importance in achieving personalized and predictive healthcare promise.

The manuscript critically assesses and aligns the integration of advanced technologies, such as quantum intelligence, with federated learning and Gen AI, within a secure and safe PHM ecosystem. It recognizes the considerable opportunities this programme offers for accelerating citizen diagnostics and tailoring treatments to improve population health outcomes. However, it equally stresses the inherent difficulties related to data privacy, algorithmic bias, and the necessity for explainable AI to build confidence for clinical uptake. Trusted Research Environments with classification points to a mechanism for enabling secure data analysis and collaboration while protecting patient confidentiality.

Section 4.4 debates the strategic considerations for expanding national ecosystem safe spaces and ensuring continuous improvement in developing and adopting BM classifications. It extends this discussion internationally, examining the potential for global collaboration in PHM development and the uptake of a United Nations HPO policy. The manuscript balances the optimistic view of technological progress with a realistic awareness of the regulatory, ethical, and logistical obstacles while addressing them to ensure equitable and safe implementation, advocating for stewarding a cycle of continuous improvement.

Realizing this vision depends on sustained investment in infrastructure, research, and workforce stewardship on classifications. Establishing clear and adaptable regulatory structures support innovation while protecting patient interests. Building public trust through transparent communication and proactively addressing ethical concerns will be crucial for widespread acceptance. Pursuing international collaboration and working toward global standards in data sharing for ontology will amplify the impact of these advancements, positioning the UK as a leader in this transformative field for improved health outcomes. Stewarding biological classifications with HEMSS requires an appropriate HPO policy that mission populace health cooperatively.
